# Breastfeeding and infant care as ‘sexed’ care work: reconsideration of the three Rs to enable women’s rights, economic empowerment, nutrition and health

**DOI:** 10.3389/fpubh.2023.1181229

**Published:** 2023-10-11

**Authors:** Karleen D. Gribble, Julie P. Smith, Tine Gammeltoft, Valerie Ulep, Penelope Van Esterik, Lyn Craig, Catherine Pereira-Kotze, Deepta Chopra, Adiatma Y. M. Siregar, Mohammad Hajizadeh, Roger Mathisen

**Affiliations:** ^1^School of Nursing and Midwifery, Western Sydney University, Parramatta, NSW, Australia; ^2^National Centre for Epidemiology and Population Health, Australian National University, Canberra, ACT, Australia; ^3^Department of Anthropology, University of Copenhagen, Copenhagen, Denmark; ^4^Philippine Institute for Development Studies, Quezon City, Philippines; ^5^Department of Anthropology, York University, Toronto, ON, Canada; ^6^Department of Sociology and Anthropology, University of Guelph, Guelph, ON, Canada; ^7^School of Social and Political Sciences, University of Melbourne, Melbourne, VIC, Australia; ^8^School of Public Health, Faculty of Community and Health Sciences, University of the Western Cape, Cape Town, South Africa; ^9^Institute of Development Studies, University of Sussex, Brighton, United Kingdom; ^10^Center for Economics and Development Studies, Department of Economics, Faculty of Economics and Business, Universitas Padjadjaran, Bandung, Indonesia; ^11^School of Health Administration, Dalhousie University, Halifax, NS, Canada; ^12^Alive and Thrive East Asia Pacific, FHI Solutions, Hanoi, Vietnam

**Keywords:** gender equality, breastfeeding, maternal nutrition, care economy, maternity leave, maternity protection, national accounting

## Abstract

Women’s^1^ lifelong health and nutrition status is intricately related to their reproductive history, including the number and spacing of their pregnancies and births, and for how long and how intensively they breastfeed their children. In turn, women’s reproductive biology is closely linked to their social roles and situation, including regarding economic disadvantage and disproportionate unpaid work. *Recognizing*, as well as *reducing* and *redistributing* women’s care and domestic work (known as the ‘Three Rs’), is an established framework for addressing women’s inequitable unpaid care work. However, the care work of breastfeeding presents a dilemma, and is even a divisive issue, for advocates of women’s empowerment, because reducing breastfeeding and replacing it with commercial milk formula risks harming women’s and children’s health. It is therefore necessary for the interaction between women’s reproductive biology and infant care role to be recognized in order to support women’s human rights and enable governments to implement economic, employment and other policies to empower women. In this paper, we argue that breastfeeding–like childbirth–is reproductive work that should not be reduced and cannot sensibly be directly redistributed to fathers or others. Rather, we contend that the Three Rs agenda should be reconceptualized to isolate breastfeeding as ‘sexed’ care work that should be *supported* rather than reduced with action taken to *avoid undermining* breastfeeding. This means that initiatives toward gender equality should be assessed against their impact on women’s ability to breastfeed. With this reconceptualization, adjustments are also needed to key global economic institutions and national statistical systems to appropriately recognize the value of this work. Additional structural supports such as maternity protection and childcare are needed to ensure that childbearing and breastfeeding do not disadvantage women amidst efforts to reduce gender pay gaps and gender economic inequality. Distinct policy interventions are also required to facilitate fathers’ engagement in enabling and supporting breastfeeding through sharing the other unpaid care work associated with parents’ time-consuming care responsibilities, for both infants and young children and related household work.

## Introduction

Breastfeeding, the process by which infants and young children suckle and receive nourishment and comfort at the human breast, is a type of care work that has profound effects, not only on child health, survival and development, but also on the health and well-being of women (1). Care work consists of activities and relations needed to fulfil physical, psychological and emotional needs of people, including infants and young children, older adults, the sick, and the disabled (2). Caring activities can be direct (e.g., feeding an infant, caring for a sick person) or indirect (e.g., cleaning, cooking) (2). According to the International Labor Organization (ILO), *‘care work is at the heart of humanity, as all human beings are dependent on care to survive and thrive.’* Not appropriately supporting unpaid care work weakens the social capacity on which societies depend (3–5). While unpaid care work is vital, women disproportionately undertake it, which diminishes their well-being and hampers their empowerment and full participation in society (6). We hold the position that care work has a *‘widespread, long-term, positive impact on wellbeing and development’* (7), and that the problem is not care itself*, ‘but the fact that care… is disproportionately performed by women’* (8). It is recognized that the sex disparity in unpaid care work must be addressed in order to achieve the Sustainable Development Goal (SDG) to empower women and girls (9). However, the nature of breastfeeding as a part of female reproductive biology and resultant interactions between women’s biology and health, their caregiving, and societal structures and expectations must be accounted for in initiatives to empower them (10). Where they are not, actions intended to promote gender equality can have the effect of undermining women’s human rights, including their rights to health and to work, with resultant serious adverse consequences for women, children, and society.

Breastfeeding children for 2 years or more, exclusively so for the first 6 months, is recommended by the World Health Organization (WHO) and UNICEF (11). Undermining the care work of breastfeeding specifically risks harm to women’s reproductive health and nutrition. For example, closely spaced births (<24 months) make women more vulnerable to anemia (12–14), the most common nutritional deficiency in women of reproductive age affecting 40% of pregnant women globally (15). However, exclusive breastfeeding for the first 6 months of a child’s life powerfully suppresses ovulation and so is highly protective against early pregnancy (16) and breastfeeding beyond this time continues to impede conception. In Nigeria, breastfeeding for 10 or more months was strongly associated with an interbirth interval of more than 24 months (17). In Jordan, women who breastfed for more than 12 months had an interbirth interval 5 months longer than women who breastfed for a shorter time (18). Fertility suppression due to breastfeeding is stronger for women who are poorly nourished so providing additional protection to those who need it most (19). A recent study in sub-Saharan Africa, confirmed the importance of breastfeeding for fertility regulation among the poorest women (20). Where women are so severely malnourished as to compromise lactation, improving maternal nutrition status with supplementary foods is less costly and more effective than supplying inferior infant formula for the child (21, 22). In other words, feed the mother and not only will that improve her nutritional status and health, but she can also feed her baby. However, nutrition for breastfeeding women is often not given the attention it deserves (23–25). Elevated rates of maternal morbidity and mortality as well as poorer infant outcomes across all contexts are associated with short interpregnancy intervals, even in high resource contexts (26).

Reproductive biology, including related to premature cessation of breastfeeding, is also at the center of the high incidence of reproductive cancers experienced by women in high-income countries (HIC) (27). Research conducted in 2002 using data from 57,000 women worldwide, suggested that rates of breast cancer in HIC are reduced by 42% where women breastfed each of their children for the recommended duration (28). Epidemiological evidence reviews further show that women who cease breastfeeding early are at increased risk not only of breast and ovarian cancers but also of other non-communicable illness like diabetes and heart disease (29–31). Globally, it has been calculated that at least 845,000 annual deaths of children (largely from infectious disease and malnutrition) and 98,000 maternal deaths (from breast cancer, ovarian cancer and type II diabetes) are attributable to premature breastfeeding cessation (1, 31).

### The three Rs: improving equality with respect to breastfeeding

The Three R’s: Recognize, Reduce and Redistribute, form the basis of an influential framework for addressing the disproportionate unpaid care work that hampers women’s equality. The first component, recognize, involves ensuring that care work is valued and appreciated as vital to societal well-being (6). Once recognized, it is understood that measures should be taken to reduce (2nd R) and redistribute (3rd R) this care work to others including male partners, other family members, employers, and the state so that women are no longer bearing a disproportionate care responsibility (6). Reduction and redistribution of women’s child caregiving work is considered by some to be particularly valuable because it can facilitate maternal engagement in the paid workforce and thus, it is argued, economic security and empowerment (32, 33). Many imply or explicitly state that caregiving of infants should be included in the redistribution of care work and that sex equity in the care of infants is desirable [e.g., (34–37)].

Breastfeeding poses a challenge to child-care egalitarianism (33, 38). National time-use surveys, which collect time-diary data on all activities people do over a 24-h day, show that breastfeeding is time intensive. Australian research found that women who were exclusively breastfeeding infants 3–6 months of age spent an average of 17 h a week breastfeeding with an additional 11–12 h of associated emotional care (soothing, holding or cuddling) (39). Breastfeeds are spread over the day and night; the WHO Multicenter Growth Reference Study found that exclusively or predominantly breastfed 3 month old infants in Brazil, Ghana, India, Norway, Oman, and the United States breastfed a median of 10 times per day with some feeding in excess of 15 times (40). Breastfeeding is therefore more difficult whenever infants are not proximate to their mothers, including due to paid employment.

Women in the paid workforce in Zimbabwe were found to be prevented from breastfeeding on demand (41) which, due to the supply–demand process of breastmilk production (42), would jeopardize their breastmilk supply. It is therefore unsurprising that mothers in the paid workforce in Brazil (43), Zimbabwe (41), and Nepal (44) were more likely to prematurely cease exclusive breastfeeding than those not in paid employment. Employment also has an impact on women’s ability to continue any breastfeeding; in Ethiopia, employed women were less likely to be breastfeeding their infants at 1 year than women who were not employed (45). In India and South Africa, despite good knowledge about the importance of breastfeeding, women in the informal workforce stopped breastfeeding after work return because of logistical and social difficulties (such as breastfeeding in public) (46). And in the United States (47) (where there is no legislated paid maternity leave) and Australia (48) (where there is entitlement to a short paid maternity leave), women commonly nominate returning to work as a reason for stopping breastfeeding. Unsurprisingly, given the constraining impact of maternal employment on breastfeeding, there are implications for infant health with maternal employment. Studies from Pakistan (49), Bangladesh (49), Ethiopia (45), India (49) and Nepal (44, 49) have found maternal paid employment to be associated with higher child malnutrition rates.

While it is established that women’s reproductive rights around pregnancy should be supported, it is often overlooked that breastfeeding is also a part of female reproduction. Lactation begins with the production of colostrum during pregnancy, and onset of copious milk secretion follows the removal of the placenta after birth (50). Breastfeeding is initiated with the child first suckling at the breast and may continue for some years. Pregnancy, birth, and breastfeeding form a reproductive continuum and breastfeeding is a biological process that has reproductive health implications and reproductive rights associated with it (38, 50, 51). However, breastfeeding is both a biological and social phenomenon. Social phenomena (structures, institutions, rules) influence breastfeeding through how they deal with the biological imperative requiring time and proximity of mother and child to maintain adequate milk supply and breastfeeding duration. In recognition of this, women’s breastfeeding rights are supported by a number of human rights instruments and related documents [e.g., (52–60)]. Women having human rights in relation to breastfeeding does not mean they are compelled to breastfeed, any more than they are compelled to become pregnant or to use contraception. It also does not mean that breastfeeding is easy or desired by every woman. Rather, it means that women should not be prevented from breastfeeding and are entitled to proper health support, protection from misinformation on infant and young child feeding, and family and societal support so that they are able to breastfeed (50, 61).

### Paid maternity leave as a support for women’s health and reproductive right to breastfeed

While some explicitly position breastfeeding as a barrier to women’s equality (33), others have called for a more appropriate consideration of women’s breastfeeding and infant and young child care work in policy (62). Paid maternity leave provides critical support for women’s childbirth recovery and breastfeeding and was prioritized by the ILO for inclusion in the first Maternity Protection Convention adopted in 1919 (38). Women’s rights to maternity leave and breastfeeding breaks in that early Convention have subsequently been updated, and elaborated in other broader human rights instruments related to women’s health, and to non-discrimination in employment (38). It is recognized that in order for paid maternity leave to support the rights of all women, it needs to be available to women not only in the formal sector, but also in the informal sector, where most women work (63).

Availability of paid maternity leave has measurable impacts on breastfeeding rates. Analysis of Demographic Health Survey (DHS) data from 38 low- and middle-income countries (LMIC) found that a 1 month increase in paid maternity leave was associated with a 7.4 percentage point increase in early initiation of breastfeeding, a 5.9 percentage point increase in exclusive breastfeeding of infants 0–5 months, and a 2.2 month increase in average breastfeeding duration (64). Expanding paid maternity leave in Canada from 6 to 12 months increased rates of exclusive breastfeeding by 40% and breastfeeding duration by 1 month on average as well as decreasing the proportion of women who said they stopped breastfeeding because of work (65).

Paid maternity leave also protects maternal short- and long-term health. In Australia, the 2011 introduction of 18 weeks paid parental leave improved the short-term mental and physical health of women, especially those in insecure casual employment (66). In Norway, the 1977 introduction of 4 months paid maternity leave (and 12 months unpaid leave) improved health metrics for women at age 40 years including body mass index, blood pressure, pain, and mental health (67). It was also associated with women taking less sick leave for breast and ovarian cancers (67). Single and socioeconomically disadvantaged mothers benefitted most. Given the known impact of breastfeeding on women’s health, these outcomes may have been related to more mothers being able to breastfeed for longer. In Europe, paid maternity leave of 12+ weeks at first birth was associated with lower depression scores when women were 50+ years old (68). A recent systematic review found that women’s mental health is consistently improved by maternity leave access and the more generous the leave in duration and payment, the greater the benefit (69).

Access to maternity leave also supports infant health and development. Analysis of DHS data from 20 LMIC found that each additional month of paid maternity leave was associated with 7.9 fewer infant deaths per 1,000 live births (13% relative reduction) (70). Each week increase in maternity leave also improved the rate of the 3^rd^ diphtheria, tetanus, pertussis vaccination by 2.2 percentage points (71). In the United States, infants of women who accessed unpaid maternity leave had almost half (47%) the risk of being hospitalized as compared to infants of mothers who did not access maternity leave (72). Infants of university educated, married women who took unpaid maternity leave were 16% less likely to die from congenital abnormalities or undefined causes (such as Sudden Infant Death Syndrome) compared to infants of demographically similar women who did not take maternity leave (72).Tanaka (73) looked at the impact of post-birth paid leave on infant mortality in 18 high-income countries and found that each 10 week increase in leave was associated with a 4.1% decrease in post-neonatal mortality. These findings suggest that in addition to benefitting maternal health, maternity leave is protective of infant health, and this is the case from the poorest to the wealthiest contexts.

Although maternity protection policies and programs are fundamental to promoting maternal and infant health and nutrition as well as gender equality, inadequate access to maternity leave remains an issue (74). The most recent ILO global review found only 42 (out of 185) countries provided 18 weeks or more paid maternity leave (75). However, research costing expansion of paid maternity leave in Brazil, Ghana, and Mexico found that increasing leave by 1 week would have a median cost of US$195 in Brazil, US$50 in Ghana, and US$83 in Mexico (76). These amounts are not large in comparison to the cost of out-sourced childcare and infant formula; in Mexico, the per child cost of social security day-care services is US$56 and the weekly cost of infant formula is US$39 (76). Recent research by the ILO found that implementing comprehensive and adequately paid maternity and parental leave and providing breastfeeding breaks at minimum standards is economically feasible in all country settings with a global cost in 2030 of US$269 billion, being <0.5% of Gross Domestic Product (GDP) (77). This is lower than estimates of the annual economic costs of not breastfeeding of US$341.3 billion (1) to US$570 billion (78).

Allowing fathers to take paternity leave simultaneous with mothers supports women’s breastfeeding and their health. In Sweden, fathers taking leave at the same time as mothers was associated with a longer duration of exclusive breastfeeding (79). Also in Sweden, provision for fathers to take intermittent leave alongside mothers, including as single days, resulted in a 38% reduction in maternal inpatient or outpatient treatment for childbirth complications, 31% reduction in likelihood of an antibiotic prescription, and a 45% reduction in prescription of anti-anxiety medication in the first 6 months of a child’s life (80). This suggests flexibility for parents to tailor leave-taking, has health benefits for women and children. However, at the present time, while many HIC have paid paternity measured in weeks to months, most LMIC have no to less than a week of such leave (81).

### Breastfeeding as ‘sexed’ care work

Where government policy allows either mothers or fathers to take leave after a child’s birth, virtually all mothers take all leave available to them. In Finland, only 1% of women do not take the entirety of maternity leave reserved solely for them as well as all of the parental leave available to either parent (34). And in Spain, mothers take 99.5% of parental leave (34). However, the fact that women rather than men overwhelmingly take workplace leave after a child’s birth, has been positioned as *‘undermining the gender equality project’* (33). The UN-supported campaign organization MenCare advocates that men and women each be provided with 16 weeks paid, non-transferable, parental leave after the birth of a child (37). They argue that that gender equality requires that men undertake 50% of child caregiving with no exception for infants and no mention or consideration of breastfeeding (37). In this view, parental leave is conceptualized as a tool for reducing women’s unpaid care work via promoting sex equity for infant care rather than as societal support for the reproductive and work rights of women, and right to health of children (38, 51, 82). This line of reasoning, whether knowingly or not, positions infant formula as providing women with *‘liberation in a can’* (51), without consideration of the cost to the health or rights of women and children. This logic also impinges upon women’s agency and promotes actions to reduce women’s leave taking for their own good. This impingement is not accidental, with opinion leaders being explicit that *‘imposing significant restraints on women’s choices,’* is justified to achieve gender equality (83, 84). In practice, this means coercing women to reduce their desired participation in infant care and breastfeeding.

Holding sex equity for infant care as a goal warps policy assessments of countries’ gender equality measures. For example, the United States, with no legislated paid maternity leave (85), was ranked 11th out of 21 high income countries for gender equality in one parental leave policy assessment largely because mothers and fathers were equally bereft of paid leave after an infant’s birth (82). This ranking was above countries with 6 or more months of paid maternity leave and seemed oblivious to the real-life impact lack of paid maternity leave has on the lives of American women and children. A significant proportion of women in the United States return to work within 6 weeks of giving birth (86).

Some argue that it is men’s reluctance to take leave that creates the imbalance between mothers’ and fathers’ leave-taking after a child’s birth (34). However, this is contrary to evidence describing women’s agency and reasons for leave taking. Research from Iceland found it was women who decided which parent took leave after a child’s birth (33). Desiring breastfeeding continuance is frequently nominated by women as why they take all paid leave available to them (33, 87) and also for taking additional unpaid leave (88). Some high-income countries, have instituted so called ‘daddy quotas’ of ‘use-it-or-lose-it’ paternity leave which cannot be transferred to mothers in order to leverage women back to work sooner and fathers into infant care (36). However, in Sweden the non-transferable daddy quota has resulted in women ceasing breastfeeding before they wished when their partner takes on primary infant care and they return to work (89). In Norway, women expressed dissatisfaction with the amount of paid leave available to their partners because they wanted more leave for themselves (88). Despite policymakers’ attempts, women (on average) are persistent in seeking to care for their infants. Thus, in Sweden, a tax bonus for parents who share parental leave equally made no significant change to fathers’ leave taking (32). And an increase in the daddy quota in Norway and concomitant reduction in paid leave available to mothers, was associated with an increase in women taking unpaid leave despite the negative financial impact upon them (88). Time-use data shows that women’s childcare motivation remains even when they have returned to work and they may go to considerable lengths to avoid trading off paid work and child caregiving against each other, including by forgoing sleep, self-care and leisure (90).

Physiological factors at least partly underpin women’s infant-care motivation. During pregnancy, biological processes mediate structural and functional brain changes that prime women to bond to their infants and to experience pleasure in interacting with them (91). Post-birth, skin-to-skin contact with their infant and breastfeeding enable affectionate dyadic interaction and stimulate the release of the hormone oxytocin further promoting social responsiveness and maternal behaviors, including infant proximity seeking (92–98). The suggestion by some that women do not have a biological predisposition toward caring for infants they have gestated and birthed [e.g., (99)] is not borne out by the scientific evidence. However, none of this is to suggest that women should want to or must take maternity leave, to deny that social factors and societal expectations also play a role (100) or to suggest that these influences apply to caregiving more generally (including indirect care like housework (101)). Rather, this elucidates that maternal infant care motivation is influenced by female physiology and it should not be a surprise that maternity leave is valued.

The processes that support maternal caregiving can be at least partly interrupted by absence of skin-to-skin contact after birth, caring for infants in hospital nurseries rather than rooming-in, and discouraging breastfeeding (102). These interventions will reduce women’s infant care motivation, but reduced breastfeeding has adverse health consequences and reduces maternal sensitivity (1, 103). In addition, separating mothers from their infants, particularly in the hours and days after birth, impedes mother-infant attachment and maternal sensitivity (97, 104). In vulnerable mothers, early separation from infants and reduced breastfeeding may undermine their caregiving such that they abandon or neglect their children (105–109). The role of breastfeeding underpinning maternal infant care motivation was demonstrated in American research which showed that institution of breastfeeding supportive hospital policies resulted in not only an increase in breastfeeding rates and time mothers invested in child care but also an ‘unintended consequence’ of reduction in maternal labor force participation (110). Thus, the scientific evidence indicates that interventions that support breastfeeding and maternal and infant health, conflict with policies to encourage new mothers to return to paid employment during their child’s first year.

The literature describes caregiving work as being ‘gendered,’ that is women undertake more of it (paid and unpaid) than men (77). The assumption is that this disproportionate care work by women is a result of sex stereotypes about what work is appropriate for men and women and other pressures flowing from sexism. However, this understanding does not appropriately consider childbirth recovery or the complex physiology of lactation and breastfeeding. The described impact of parental leave constructions reducing women’s infant caregiving and breastfeeding, illustrate this problem. We argue that like pregnancy and childbirth, breastfeeding and the direct care of infants should not be considered ‘gendered’ care work but instead conceptualized as ‘sexed’ care work- that is work that is a part of the female reproductive continuum. This would enable a proper value to be placed on breastfeeding as a reproductive right to be protected and supported rather than something that should be reduced in the name of gender equality. It shifts the emphasis from reduction and redistribution of breastfeeding and infant care to recognition and support. It allows an understanding that women need ‘substantive equality’ that reflects they bear and breastfeed children rather than ‘formal equality’ that does not see sex difference (111–113). Schoenbaum and Fontana (112) and Schoenbaum (113) provide arguments for applying formal equality to pregnancy and breastfeeding. However, achieving substantive gender equality requires women’s and children’s breastfeeding rights not be undermined but enabled, by the father, their family, society, and the state meeting their respective duties to care for and support the child and the mother during the breastfeeding period.

### Use-it-or-lose-it paid paternity leave only marginally increases men’s unpaid care uptake

Use-it-or-lose-it paid paternity leave is commonly advocated for in order to increase gender equality [e.g., (36, 37, 114)]. The underlying belief is that involving fathers in infant care early is valuable for increasing father-child bonding and care competence and inducing long-term greater childcare and domestic work involvement (35, 36). In this way, non-transferable paternity leave could redistribute care work to men and reduce women’s unpaid childcare and domestic work. Although as previously described, use-it-or-lose-it paternity leave can have the effect of reducing breastfeeding if maternity leave is not sufficiently long, some might argue this could be justified if it substantially increased gender equality. While use-it-or-lose-it paternity leave successfully induces more fathers to take leave (33), the impact on fathers’ unpaid care work uptake or gender equality is questionable.

In Sweden, the 1995 introduction of 1 month non-transferable paid paternity leave did not have any effect on fathers’ propensity to care for a sick child (a measure of parenting involvement) or on wages or employment rates of mothers and fathers (114). In Germany, the 2007 institution of 2 months paid paternity leave resulted in fathers reporting they spent an average 36 min more per weekday on childcare in their child’s first year and 26 min more per weekday on childcare when their child was 18 and 30 months old (115). However, these changes were in the context of mothers spending an average of more than 10 h per day on childcare while fathers spent a quarter of this time so engaged (115). The daddy quota had no impact on the time fathers spent on housework (115). Bunning et al. (116) found that German fathers who took paternity leave, self-reported more engagement with childcare and reduced paid work hours regardless of their leave length or whether they took it simultaneous with mothers. Fathers who took more than 2 months of leave or who took solo leave reported more time spent on housework (116). Unfortunately, comparison to women’s time spent on childcare and domestic work was not made. Other research in Germany, found that men who took any paternity leave self-reported an increase in childcare and housework 4 years after their child’s birth but employed mothers were still responsible for 77% of household time spent on housework and 73% of childcare time (117). In Norway, Cools et al. (117) found the 1993 introduction of 1 month use-it-or-lose-it paternity leave (typically taken when leave available to women was used up 10 months after birth) did not alter men and women’s relative engagement in paid employment (117). Kotsadam and Fineraas (118) found reduced reported conflict over household work and reportedly more male involvement in clothes washing (but not other housework) when children born immediately after Norwegian paternity leave was instituted were 10–12 years old, but did not distinguish between men and women’s perceptions of change.

Thus, reported changes in men’s childcare and domestic work in response to non-transferable paid paternity leave are small in comparison to the amount of unpaid care work women undertake, even for long and solo paternity leave. In addition, all research on the impact of use-it-or-lose-it paternity leave is based on stylized estimates of activities, which calls into question the accuracy of any improvement findings (119). Men consistently over-report their time spent on domestic work whereas women under report (120). In societies that value gender equality, self-reported estimates of fathers’ unpaid care work are likely to be influenced by motivation to provide socially desirable responses (121). Data collected using time-use diaries is more accurate (121), but time-use research on the impact of paid paternity leave on men’s engagement in childcare and domestic work is lacking.

However, time-use studies provide insight into differences in how men and women undertake childcare and other care work. They show measurement of caregiving work purely on an hourly basis underestimates women’s work and the impact this work has on their lives as compared to men. Australian research considering parenting of young children showed that half the time fathers spent caring for children, they were only ‘looking after’ them rather than performing active care tasks, and 90% of time fathers were with their children, their spouse was also present (122). A high proportion of men’s childcare is in child enrichment activities such as playing or reading to children as compared to physical care (e.g., washing, dressing, feeding) whereas women have the opposite pattern (122, 123). Child enrichment activities are arguably more pleasant, and as they are usually not time sensitive, more easily fitted in around other activities, compared to care work that is timetabled like dressing or feeding children or transporting them to and from external care (122). Providing child enrichment care is therefore less costly in terms of impact on other activities including paid employment. This same research found that employed mothers spent twice as much time performing childcare (as a primary or secondary activity) as employed fathers did, with mothers multitasking much more often (122). In this way, mothers preserved time interacting with their children by undertaking greater task density, meaning they worked harder than fathers and undertook more work in the time available to them (122). An analysis of Australian time-dairy data of childcare and domestic work considering primary and secondary activities found that excluding secondary activity from the analyzes underestimated the workload of mothers of pre-schoolers by 90% (124). Taken together, this research calls into question the veracity of claims regarding significance of any increased childcare and domestic work as a result of paid paternity leave.

The impact on breastfeeding of societal expectations that mothers and fathers should share infant care equally, such as in Scandinavia, should also be considered. In Sweden, parenting handbooks position breastfeeding as a ‘gender equality problem,’ and describe breastfeeding as alienating fathers because it distances them from newborns and makes them unimportant (125). Research from Norway found that some fathers felt jealous of the mother–child relationship and sad and excluded when women breastfed (126). They describe breastfeeding as positive for children but negative for them. While one way of coping with this exclusion was doing more non-infant care work, another way was withdrawing from their partner and child (126). It is noted that breastfeeding as an embodied and sexed practice presents a barrier to societal ambitions for sex equity in child caregiving (126). Given the importance of partner support for breastfeeding exclusivity and continuance (127), promotion of the idea that fathers should be equally involved in infant care may undermine breastfeeding. Communicating with fathers about the special nature of the sexed care work of breastfeeding and their important role in supporting breastfeeding and undertaking other care work may assist them in adjusting to fatherhood and increase their other care work and breastfeeding rates (128, 129). This is an area where further research is needed.

Where fathers taking paternity leave results in breastfeeding mothers returning to work, women need to breastfeed or express and store breastmilk at work. The ILO Maternity Protection Convention requires paid breastfeeding breaks and research shows they improve breastfeeding rates (130). However, large numbers of women globally do not have access to paid breastfeeding breaks (130) so extending unpaid care work into the workplace. In addition, facilities to express and store breastmilk in workplaces or nearby childcare is not available to many women, increasing the difficulty of breastfeeding continuance (131).

In summary, there is not strong evidence that paid paternity leave significantly increases men’s propensity to undertake childcare or domestic work or meaningfully reduces women’s unpaid care work. Nor does it seem that reducing the gap between men and women’s parental leave entitlements consistently impacts the pay gap between them, women’s career progression, or influences workforce participation (81). For example, a negative association has been found between the gap in maternal and paternal leave entitlements and the female labor force participation in the East Asia Pacific, South Asia, and Sub-Saharan Africa regions (81). However, the opposite is the case in the Middle East and North African regions and there no relationship between the parental leave gap and female workforce participation in other global regions (81). This reflects the importance of context on the impact of interventions.

Parental leave constructions that leverage men into paternity leave and women back to work earlier, may simply worsen women’s situation as they undertake the ‘second shift’ of childcare and household work, in addition to paid employment sooner (132). We therefore argue that parental leave should focus on supporting women’s reproductive rights and children’s rights to the highest attainable standard of health. As described, evidence suggests that this is enabled where women have access to long parental leave, where reserved paternity leave is in addition to this leave, and where any reserved leave for fathers can be taken intermittently and simultaneous with mothers. Some have expressed concern that generous leave entitlements for new mothers may result in long-term adverse employment disadvantage for women. Globally, becoming a mother has a negative impact on women’s short and long-term earnings. However, there is not a straightforward relationship between leave available to mothers and women’s long-term earnings. Organization for Economic Co-operation and Development (OECD) data shows that countries like Sweden and Norway with generous paid leave for new mothers have a lower overall gender pay gap than the US and Switzerland (which have no and relatively short maternity leave respectively) (133). Kleven et al. (134) found that Sweden and Denmark have a smaller long-term pay gap for mothers as compared to the United States. They conclude that leave policies do not underlie the pay gap between parents.

Thus, fathers should be supported through means other than leave incentives at the expense of women to do more of the gendered care tasks such as household work, care of older children and infant care around breastfeeding. As found in Australian research, fathers’ work hour length may have more impact on propensity to engage in activities like child care than taking leave after the birth of an infant (122, 135). Broader social change is needed to properly support care work, including breastfeeding, and enable gender equality.

### Recognizing breastfeeding as economically important care work

The sexed care work of breastfeeding is critically important to societies and yet is scarcely visible or valued (136). Appropriately recognizing and valuing this work requires that it be measured. Time-use data assists in making visible to policymakers the importance of unpaid care work as an underpinning of the monetary economy (3) and provides a more complete picture of a country’s economic activities (137). It also makes it possible to relate the value of unpaid care work to market-focused economic statistics and for it to be accounted for in monetary terms so enabling factoring into policy development (138–140). However, traditional time-use surveys do not adequately capture women’s breastfeeding and infant care work (141). Australian research found that women’s overnight infant care, secondary care (care undertaken when doing another task), and 11 h per week of breastfeeding were not captured in conventional time-diary analyzes which tend to consider ‘childcare’ as an aggregate and/or focus on main or ‘primary’ activities (142). They fail to distinguish time spent breastfeeding and caring for infants from time spent simultaneously caring for older children or undertaking other tasks, so obscuring the work of breastfeeding (143). In order for time-use data to accurately reflect the work of breastfeeding and infant care, the full potential of time-use surveys must be used to account for and separate out multiple tasks within childcare and other care work, including breastfeeding (142).

Lack of reliable time-use data is just one of the barriers to incorporating unpaid care work into measures of countries’ economies (144, 145). GDP is the standard measure used to assess national economic performance and growth. However, economic activity in GDP is defined in ways that exclude or marginalize unpaid care work (146). Exclusion of breastfeeding, and in particular the exclusion of breastmilk, from counting in GDP, is archetypal of this failing (138, 147, 148). Some progress toward inclusion of unpaid care work in economic statistics has been made by provision for satellite accounting in the foremost standard for economic activity measurement, the United Nations System of National Accounts (149). Satellite accounts provide a framework for considering the value of specific aspects of economies, including those that do not involve monetary transactions (150). Satellite accounting allows value to be placed on unpaid care work in a way consistent with national accounts, so permitting comparison with market-based activity (150). However, governments need to be motivated to accurately measure unpaid care work, to apply satellite accounting to this work, and to include this work in assessments of economic progress.

It is recognized by economic experts that breastmilk meets the criteria for measurement in GDP (138, 147, 148) and if it were included, its monetary value would be substantial (151). Norway adopted the practice of counting breastmilk production in its food systems in the 1990s and continues to do so (152). However, Norway is unique in this, the rest of the world fails to count human milk production in any economic measure despite the ease with which it could be done. Not including human milk specifically in GDP, means that a decline in breastfeeding rates and concomitant increased infant formula sales results in an increase in GDP (151) wrongly suggesting economic progress. It also results in market work, including medical treatment of excess illness as a result of lack of breastfeeding, being favored over the economically valuable, but unpaid, care work of breastfeeding (153). As the previous example suggests, the economic and other costs of the loss of breastfeeding to women’s and children’s health needs also to be measured and included in economic evaluations (151, 153). The recently deployed Mothers’ Milk Tool is designed to support valuing breastfeeding (154). It allows anyone from policymakers to mothers themselves to calculate the monetary value of breastmilk, and the cost of the ‘lost milk’ when mothers are not enabled to breastfeed (78), but this is just a start.

Alternative measurements of societal health focusing on well-being, are being trailed in some countries and sub-jurisdictions and have potential to reduce the harms from policymaking centered on GDP (155, 156). However, the existing and proposed well-being frameworks give little attention to unpaid work and are unlikely to challenge the deeply flawed paradigm which underpins current accounting measures (144). Challenging the failure of conventional and novel economic accounting systems to measure breastfeeding women’s productivity is needed to transform economic institutions that currently disadvantage women (157). A recent publication by the WHO Council on the Economics of Health for All noted that *‘pregnancy, childbirth and lactation are at the center of health for all, but since human reproduction is women’s work, these activities do not count’* (158). This must change.

### Paid employment, women’s empowerment, childcare, and child development

As previously stated, reduction and redistribution of women’s unpaid childcare work is often considered valuable as it is purported to enable women’s empowerment *via* paid employment. However, leading economists flagged nearly a decade ago that policies based on the idea that women’s inequality can be solved by workforce opportunities alone will fail if the proposed solution of more paid work intensifies the problem of insufficient time for essential care work, including breastfeeding, and for greater equality in leisure (159–161). Indeed, time-use data for selected high income countries suggests there has been declining leisure time for women alongside changing roles in childcare and female workforce participation in contrast to increasing leisure for men (143). It is clear that rather than paid employment being empowering by its very nature, women’s empowerment requires quality paid work and a positive balance between paid work and unpaid work including childcare (162, 163).

Not factoring the dynamics of women’s unpaid care work into programs to promote women’s empowerment via paid employment can engrain inequalities. For example, India’s Mahatma Gandhi National Rural Employment Guarantee Act (MGNREGA) seeks the empowerment of the disadvantaged, especially women, through employment in building community infrastructure (163). Initial proposals for the MGNRGA included childcare for young children at any worksite where there were 20 or more women with some arguing that creches be established where there were 10 or more workers of either sex. However, childcare provision was not a priority of those with power and a more limited provision for childcare; where five or more children were present was instituted (163). The MGNREGA also lacked maternity leave, breastfeeding provisions and any measure of quality control or enforcement of childcare (163). As a result, women’s needs associated with pregnancy, birth and breastfeeding were ignored, their ability to exclusively breastfeed their infants was compromised, and they faced health difficulties associated with infrequent breast emptying (164). Their children also did not receive appropriate care (163), and for example, young breastfed infants of participating mothers were found left at home in the care of 5 or 6 year old siblings placing them at risk of serious harm (165, 166). Similar issues have been identified in other empowerment-by-work programs [e.g., (167)]. For many women, their inability to feed and ensure adequate infant care meant they did not want to participate in the MGNREGA (164). However, coercion of women into MGNREGA work, sometimes involving physical violence, has occurred (164). New mothers with these experiences did not find the MGNREGA empowering (164).

The experience of the MGNREGA also highlights the need to ensure infants receive good quality care while mothers work. It is well recognized that for healthy development, infants and very young children need to be cared for by a small number of adults *‘who reliably care for them in a calm, attentive, affectionate matter’* (168). The WHO/UNICEF Care for Child Development Program emphasizes the need for very young children to have responsive and loving care (169). In many countries, extended family, like grandparents, support parents with childcare [e.g., (170, 171)]. For example, in South Korea 57% of infants whose mothers are in paid employment are cared for by grandparents (172). In Australia, childcare arrangements involving other family members were associated with higher rates of breastfeeding among employed new mothers (173). However, in many countries there is a move away from extended family households reducing the availability of this option [e.g., (174–176)]. And in some countries, although extended family care may be customary, circumstances may make it unavailable, for example in the urban slums of Bangladesh (177). In these situations, childcare while parents work may be undertaken by older sisters, who are removed from education to do so (177). Mothers from LMIC may breastfeed their infants during their paid maternity leave but then leave them with family in rural areas when they return to work in cities (178). The childcare crisis is perhaps most exemplified in mothers who leave behind their own infants and young children with family members in order to care for the children of the wealthy in their own or other countries (179).

Group childcare options such as creches and day care centers, are increasingly presented as a solution to meeting childcare needs and enabling maternal employment. However, it is difficult and expensive to provide the responsive, loving care that infants require in group settings (168). Infants find group childcare chronically stressful, as indicated by high cortisol secretion (168) and adverse cognitive and behavioral impacts of group childcare have been noted for infants (179–182). Evidence that it is difficult to provide good substitute care for infants is also shown in research consistently associating maternal employment in a child’s first year with poorer child development and paid maternity leave with better child development. Lucas-Thompson et al. (183) conducted a large meta-analysis of the impact of maternal paid employment on children and found that maternal employment in infancy was associated with reduced scores in formal achievement tests and behavioral problems. Le and Nguyen (184) used DHS data from 29 countries to explore the impact of increased maternity leave on the height and years of educational attainment of adults born before and after maternity leave improvements. With a sample of nearly 1,000,000 individuals, they found each week increase in paid maternity leave, increased adult height by 0.056 cm and educational attainment by 0.007 years (184). In Norway, a beneficial impact of maternity leave on child development was seen in a 2% decline in high school drop-out rates and 5–7% increase in wages at age 30 for individuals born after paid maternity leave was introduced (185). These improvements were larger for those whose mothers were less educated (185). Given that maternity leave facilitates breastfeeding, the possibility that some of the positive impact of maternal care and maternity leave on child development and achievement is a result of increased breastfeeding should not be overlooked. Observational and randomized controlled trial research has found increased breastfeeding to be associated with better cognitive development (186, 187). In Brazil, those who were breastfed for 12+ months were found not only to have a higher IQ but also 0.91 years more education and BRL$341 (US$167 at the date of data collection) more in monthly income at 30 years than those breastfed for less than 1 month (188).

The time mothers invest caring for and breastfeeding their infants is valuable (189) and consideration of the value of interventions like maternity leave should include the cost of not providing maternity leave including the cost of alternative childcare and the impact on child development. The reduced time women spend providing care for their infants when they are in paid employment should also be acknowledged in measurements of productivity gains from policies of increasing maternal labor force participation (190). Not doing so overstates GDP growth and economic progress (191), and contributes to the invisibility of depletion of women’s energies and the underappreciation of the value of their care. It needs to be recognized that the developmental vulnerabilities of infants that make providing alternative care for them so difficult, decline with age and there are other important interactions between quality of care and intensity of care that mediate the impact on children (182). We are not arguing that there is no place for group childcare for infants, but that trade-offs exist and must be recognized and managed. Where child care for infants is required, it is critical to ensure that this care is accessible, flexible and of good quality (192) and involves high levels of relational, loving, responsive, and stable care. Child carers need also to have skills in caring for breastfed infants (193).

### Increasing gender equality without undermining women’s breastfeeding rights

While paid maternity leave can support women’s health and breastfeeding rights, not all women have equal access to leave. Globally, informal employment is over 60% and in LMIC this is higher, for example 86% of employment in Africa is in the informal sector (194). Many informal workers lack access to legal and social protection including maternity protection (195) and a large group of vulnerable women face additional challenges in combining employment with unpaid care work like breastfeeding (63). The inaccessibility of maternity protection to non-standard workers has been shown to disrupt breastfeeding (196). Recently conducted costing estimates in the Philippines, Indonesia, Ghana, Mexico, and Brazil demonstrate that government cash transfer programs for women on maternity leave in the informal sector is financially feasible in LMIC with heterogenous fertility and labor market structures (76, 197–200). However, even with improved policy, much country legislation on maternity protection is inadequate, and insufficiently implemented (201). For example, South Africa has specific legislation for groups of non-standard workers, yet women find it difficult to access cash payments when on maternity leave (202). Therefore, access to comprehensive maternity protection for all female workers needs to be ensured to support the precarious trade-offs that women face between paid work and caring for and breastfeeding their infants.

Workplace arrangements can enable women to more easily combine breastfeeding and employment. This includes via breastfeeding breaks, such as for example in Turkey where women are entitled to 3 h per day of ‘breastfeeding leave’ for the first 6 months and 1.5 h for the second 6 months with night shift work not permitted until the child is 2 years old (203). Breastfeeding breaks should be accompanied by other supports including a private space to express milk or breastfeed and refrigeration facilities (204). Childcare centers close to or in the workplace (203), workplaces located in or close to home (205) and where possible, allowance for bringing infants to work are further supports for women’s breastfeeding work. The COVID-19 pandemic enabled work from home on a vast scale so allowing new mothers to breastfeed longer (206), but continuation of this practice is yet to be tested. In Nordic countries, state-mandated part-time and flexible work arrangements are valued and frequently taken up by mothers (207). However, part-time workers should not be discriminated against (208) and for example, workplace policies should not exclude women who work part-time or casually from professional development or promotion.

Evidence of a recent global systematic review of breastfeeding interventions in the workplace suggests that workplace interventions can increase the duration of breastfeeding and prevent early introduction of breastmilk substitutes (209). The shorter maternity leave is, the more important workplace accommodations are, and their absence can reasonably be considered a form of sex discrimination.

Workplace accommodations will not be sufficient to enable women to breastfeed if the workplace culture is hostile toward breastfeeding. Research highlights that support for breastfeeding mothers in the workplace is mediated by the experience, knowledge, beliefs and attitudes of co-workers and managers. This includes regarding a perception or not of unfairness for taking breastfeeding breaks and stigmatization of breastfeeding as unprofessional and breastmilk as an unclean body fluid (209, 210). Recent qualitative research in Mexico identified that enabling or impeding mechanisms for working women who were breastfeeding included the level of awareness of maternity protection legislation and actual usage of breastfeeding interventions that, in turn, depended on workplace culture and supervisor and/or co-worker support for breastfeeding (211). Previous quantitative research in Australia demonstrated a link between measures of workplace support and maintaining exclusive breastfeeding for 6 months (212).

Addressing the disproportionate unpaid care work undertaken by women has important implications for policies targeting the pay gap between men and women (213). There is evidence that taking extended maternity leave may reduce women’s perceived value in the labor market and harm them economically (214). Some of this cost can be reduced through measures that facilitate maintenance of connection between women and the workplace while they are on maternity leave as well as by communication of women’s commitment to their career despite taking leave (215). However, increasing paternity leave at the expense of maternity leave risks widening gender short-term pay gaps if mothers extend their time with the infant through taking unpaid leave (88).

Bigger moves toward societal valuing of care work, including breastfeeding, *via* measures such as the previously described economic accounting for care work in GDP are needed for the cost of this infant care to be distributed between women, men and society as a whole. The inclusion of care work in national economic measurements would likely increase government, business and societal support for this work and result in new and innovative ways of addressing gender inequality. Consideration of the value of breastfeeding as food work is often overlooked [e.g., (216)] but recognizing how human milk provides food security for infants may assist in remediation (217, 218). More broadly, addressing inequality in unpaid work may require tackling other inequalities. Gendered roles in household work and childcare may be reinforced where paid hours are excessive, and wages are low (135). Countries with greater gender equality tend to be those where policies support shorter working hours for both parents of young children (219) and this may be a prerequisite for redistribution of unpaid work by men and women within households (220).

Finally, programs to encourage fathers to support breastfeeding including by engaging in care work that is not sexed in nature, such as housework, cooking, and shopping, have been found to increase exclusive breastfeeding rates (129, 221) and some research also shows an increase in care work (222). Providing fathers with breastfeeding education and facilitating peer support for them are other ways of increasing their motivation, confidence and ability to support women in breastfeeding (223, 224). Reducing commuting times (225) and the maximum length of full-time work hours provides men with more time to engage in care work (226). This can include unlimited indirect care of infants and greater care of older children, noting that the brains of fathers and their caregiving capacity are also impacted by caring for children (227). Direct care and close physical contact with infants, including for example skin-to-skin, promotes paternal role attainment, sensitive interactions with infants and parenting confidence (227, 228). However, there is a balance to be struck and, particularly early in infancy, this should not supplant maternal care to the detriment of the sexed care work of breastfeeding. These social changes, would be to the benefit of men and women and enable a more gender equitable society while recognizing and supporting the biological elements of the feeding and direct care of infants. Of course, infrastructure to reduce the time and energy investment in caregiving such as ensuring that households have electricity and piped water can reduce overall care work demands by making caregiving easier and less time intensive (205). All initiatives to improve equality for women must involve women in policy design and ask them what they want (140); women’s empowerment *‘requires that women’s lived experiences are taken into account, especially those relating to their unpaid care responsibilities’* (163). Failure to consider the real-life experiences and wishes of women, results in policies and practices that disempower women and make life more difficult for them. Until now, policymakers have not properly recognized the sexed care work of breastfeeding, nor recognized that actions toward gender equality must exclude it from efforts of reduction or redistribution. This must be ameliorated. Gender budgeting approaches may help redress unbalanced and gender biased perspectives on fiscal priorities and taxation policy, including for social protections, investments in childcare and services and other public infrastructure, and in breastfeeding support measures such as maternity protection (147). Actions to appropriately recognize, support, and avoid undermining the sexed care work of breastfeeding are summarized in Table 1 and key actions to reduce and redistribute care work associated with infants that is not sexed in nature are summarized in Table 2.

**Table 1:** Actions to appropriately recognize, support, and avoid undermining the sexed care work of breastfeeding while redistributing the costs of breastfeeding.

**Recognize**
Undertake time-use studies of unpaid care work ensuring that time spent on secondary as well as primary childcare activities are included (i.e., multitasking) to enable women’s unpaid care work to be accurately measured
Produce satellite accounts of unpaid care work, and include breastmilk in GDP and other economic production measures
Ensure that the value of breastfeeding as food, as preventative child health and development, and as preventative women’s health is included in economic assessments and policy development
Ensure that the environmental costs of infant formula production, elevated maternal and child health care costs, elevated maternal and child mortality, and impeded child development that result from reduced breastfeeding are included in economic assessments
When assessing the value of interventions to support breastfeeding, including maternity leave, incorporate the full economic costs such as alternative childcare provision, if the intervention is not made
Spread awareness, including via public messaging, about the contribution of breastfeeding work to maternal health (including protection against closely spaced pregnancies and women’s nutrition), child health, the economy, and the wellbeing of society.
Raise awareness among employers, workers, and governments regarding the minimum requirements of comprehensive maternity protection entitlements that should be available and accessible to all working women.
**Support**
The International Labor Organization Maternity Protection Convention should be revised/updated to extend the minimum standard for provision of paid maternity leave to align with the WHO-recommended duration of 6 months of exclusive breastfeeding, as reiterated in the 2023 Lancet Breastfeeding Series ensure that cash payments during maternity leave are at 100% of previous earnings include more explicit provisions on the obligation to provide suitable childcare and paid breastfeeding breaks for at least 6 months and preferably 12 months.
Provide comprehensive maternity protection for all female workers including as appropriate: maternity leave, breastfeeding breaks, facilities for breastfeeding or expressing and storing breastmilk, working hours flexibility, part-time work options, working from home options, childcare at or nearby work, provision to take infants to work.
Educate managers at workplace settings about breastfeeding so a favorable culture can be enabled. This should also facilitate accounting for employer-based benefits such reduced absenteeism and higher morale among breastfeeding mothers.
Ensure that paid maternity leave (at 100% of previous earnings) is available and easily accessible to women in the informal sector – with suitable context-specific modalities such as via cash payments.
Apply the evidence showing that the health of women and health and development of children from maternity leave has long-term benefits, especially where paternity leave is in addition to maternity leave, and where any reserved leave for fathers can be taken intermittently and simultaneous with mothers and design parental leave on this basis.
Prioritize maternal nutrition for pregnant and lactating women, for their own health and to support their breastfeeding and infant care work
Communicate with fathers about the importance of breastfeeding, its special nature as sexed care work, and provide practical advice on meeting their critical role in supporting breastfeeding and undertaking other care work, and how this may assist them in adjusting to fatherhood
Include provision for maternity leave and childcare in work opportunities for women in vulnerable employment contexts for example migrant workers and those in development or emergency contexts.
Elevate and increase social support (including by employers) for the unpaid care work of breastfeeding and the care of young children through public messaging regarding the contribution of this work to the economy and the wellbeing of society.
Provide for reduced work hours for all parents of infants and young children, along with cash payments to provide at least a minimum level of social protection and poverty reduction
**Avoid undermining breastfeeding and redistribute the costs of breastfeeding**
In working toward gender equality, focus on substantive equality that recognizes sex difference rather than formal equality that denies women’s reproductive work and needs.
Ensure that the unpaid care work of breastfeeding and infant care is excluded from efforts to reduce and redistribute women’s unpaid care work by undertaking impact assessments of policies and interventions on the ability to carry out this work.
If it is determined that a policy or intervention may adversely impact breastfeeding, ensure adjustment to mitigate against this impact.
Design parental leave with the primary goal of supporting women’s reproductive rights as well as the rights of children to the highest attainable standard of health.
Maintain any retirement pension contributions at full time rates through maternity leave and care giving-related part-time work and ensure that women who work part-time or casually are not excluded from professional development or promotion.
Consult with women regarding their needs for support regarding their breastfeeding care work to ensure that policies and programs provide support and do not undermine in the name of gender equality.
Provide accessible and affordable childcare that is of the highest standard ensuring that childcare workers are trained in the care of breastfed infants and the storage and handling of expressed breastmilk.

**Table 2:** Key actions to reduce and redistribute care work that is not sexed in nature.

Encourage and support fathers to take on other unpaid care work, including domestic work and the direct care of older children.
Change workplace conditions to support worker’s caregiving including part-time work, work from home, and shortened work hours so that men are more easily able to undertake unpaid care work.
Provide high quality flexible work arrangements for all employees and measure their uptake.
Promote the value and status of all unpaid care work
Promote an ‘ethic of male care’ across society including in schools, the media, religious institutions, business/employers and other influential organizations in which social norms are created and reinforced.

## Conclusion

The Three Rs call for women’s unpaid care work to be recognized, reduced, and redistributed. However, the care work of breastfeeding is a special case. Its sexed nature and the breastfeeding rights held by women and children mean that this care work should be recognized but must be excluded from reduction and redistribution efforts. Rather, women’s work in caring for and breastfeeding their infants and young children should be supported and action taken to avoid undermining of women’s breastfeeding ability. Greater recognition of breastfeeding as productive work in the reproductive continuum is needed, and the incorporation of this concept into economic measurement, policy development and budgeting. Reducing the amount of direct care mothers provide to their infants and young breastfeeding children should not be a goal but reducing time and energy spent in other caregiving responsibilities should be. Doing this will contribute to women’s nutrition, health, and well-being and that of their children, while also contributing to the societies in which mothers, fathers and children live. Breastfeeding-friendly infrastructure–such as paid maternity leave, workplace breastfeeding accommodations, suitable childcare, and adequate informal support–are not only protecting the health and rights of women and children. Indeed, they also contribute significantly to societies by facilitating breastfeeding practices that help to bring healthy citizens into being. Taking a collective social approach means the economic costs of providing infant and young childcare would not fall so heavily on individual women. The specifics of how this is done will of course need to take into account country context however, in short, rather than redistributing breastfeeding and infant care, we should redistribute the cost of that caregiving. We should focus on supporting care work that is necessarily sexed, and distributing the remaining non-sexed care more equally between men and women, broader society and the state to the benefit of women, children and society as a whole.

## Author contributions

KG and RM conceived of the paper. KG wrote the first draft of the manuscript. KG, JS, TG, VU, PE, LC, CP-K, DC, AS, MH, and RM discussed and provided original content for the paper. All authors contributed to the article and approved the submitted version.
